# The resistomes of *Mycobacteroides abscessus* complex and their possible acquisition from horizontal gene transfer

**DOI:** 10.1186/s12864-022-08941-7

**Published:** 2022-10-20

**Authors:** Shay Lee Chong, Joon Liang Tan, Yun Fong Ngeow

**Affiliations:** 1 Faculty of Information Science and Technology, Multimedia University, Jalan Ayer Keroh Lama, Bukit Beruang, 75450 Melaka, Malaysia; 2 Faculty of Medicine and Health Sciences, Universiti Tunku Abdul Rahman, Bandar Sungai Long, 43000 Kajang, Selangor Malaysia; 3 Center for Research On Communincable Diseases, Universiti Tunku Abdul Rahman, Bandar Sungai Long, 43000 Kajang, Selangor Malaysia

**Keywords:** *Mycobacteroides abscessus* complex, Multidrug resistance, Horizontal gene transfer, Purifying selection, Comparative resistomes

## Abstract

**Background:**

*Mycobacteroides abscessus* complex (MABC), an emerging pathogen, causes human infections resistant to multiple antibiotics. In this study, the genome data of 1,581 MABC strains were downloaded from NCBI database for phylogenetic relatedness inference, resistance profile identification and the estimation of evolutionary pressure on resistance genes in silico.

**Results:**

From genes associated with resistance to 28 antibiotic classes, 395 putative proteins (ARPs) were identified, based on the information in two antibiotic resistance databases (CARD and ARG-ANNOT). The ARPs most frequently identified in MABC were those associated with resistance to multiple antibiotic classes, beta-lactams and aminoglycosides. After excluding ARPs that had undergone recombination, two ARPs were predicted to be under diversifying selection and 202 under purifying selection. This wide occurrence of purifying selection suggested that the diversity of commonly shared ARPs in MABC have been reduced to achieve stability. The unequal distribution of ARPs in members of the MABC could be due to horizontal gene transfer or ARPs pseudogenization events. Most (81.5%) of the ARPs were observed in the accessory genome and 72.2% ARPs were highly homologous to proteins associated with mobile genetic elements such as plasmids, prophages and viruses. On the other hand, with TBLASTN search, only 18 of the ARPs were identified as pseudogenes.

**Conclusion:**

Altogether, our results suggested an important role of horizontal gene transfer in shaping the resistome of MABC.

**Supplementary Information:**

The online version contains supplementary material available at 10.1186/s12864-022-08941-7.

## Background

Nontuberculous mycobacteria (NTM) are mycobacterial species other than *Mycobacterium tuberculosis* complex (MTBC) or *Mycobacterium leprae*. The incidence and prevalence of patients with NTM infections have dramatically increased, worldwide, in recent years [1]. In many areas, the incidence of NTM infections has surpassed that of MTBC [2]*.*

Among NTM species, *Mycobacteroides abscessus* complex (MABC) is the most important rapidly growing human pathogen capable of causing pulmonary, skin and soft tissue and disseminated infections [3]. This complex is currently divided into three subspecies, namely, *M. abscessus* subsp. *abscessus*, *M. abscessus* subsp. *massiliense* and *M. abscessus* subsp. *bolletii* [4–6]. MABC is intrinsically resistant to many classes of antibiotics available in clinical practice, including beta-lactams, rifamycin, and aminoglycosides [7]. Owing to multi-drug resistance (MDR), the treatment of MABC infection is notably difficult with no reliable antibiotic regimens [8]. This has led to MABC being described as an “antibiotic nightmare” [7].

The whole genome sequencing (WGS) technology has led to the high availability of genome sequences. Consequently, this allows the investigation of molecular determinants of antibiotic resistance in MABC. The American Thoracic Society has suggested a combination treatment regimen including macrolides (clarithromycin), aminoglycosides (amikacin) and beta-lactams (cephamycins and carbapenems) for MABC infections [9]. The therapeutic effect of the regimen has not been entirely satisfactory [8] and treatment outcomes varied across patients infected with different MABC subspecies [10–12]. However, much is still unknown about the genetics and the mechanisms of antibiotic resistance in MABC*.* In the current study, bioinformatic analyses on whole genome sequences of 1,581 MABC strains were conducted to describe their resistomes and to study the contribution of evolutionary forces to the formation of the resistomes.

## Materials and methods

### Classification

The nomenclature of *Mycobacteroides abscessus* complex (MABC) has been amended multiple times [4, 5]. The three subspecies nomenclature system is used throughout this project, namely *Mycobacteroides abscessus* subsp. *abscessus* (*M. abscessus*), *Mycobacteroides abscessus* subsp. *bolletii* (*M*. *bolletii*) and *Mycobacteroides abscessus* subsp. *massiliense* (*M. massiliense*).

### Acquisition of genome sequences

A total of 1,581 MABC genome sequences were downloaded from the National Center for Biotechnology Information (NCBI) ftp site (ftp://ftp.ncbi.nlm.nih.gov) on December 2018. The 1,581 strains were named according to respective RefSeq accession numbers for consistency throughout the data analysis. The *hsp65* from all genomes were aligned in MAFFT [13] to assess strains’ taxonomy as the members of MABC. In addition, we evaluated genome completeness of downloaded sequences against ATCC19977 by using Maximal Unique Matches index. The pairwise genome alignments were performed in MUMmer version 3.23 [14]. All the downloaded sequences were subjected for multiple analyses in 64 GB Linux OS server as illustrated in Additional file 2: Fig. S1.

### Genomes annotation and identification of orthologs

Coding sequences (CDS) and the corresponding protein sequences were achieved using GeneMarkS-2 [15]. The orthologs in MABC were clustered based on a strict definition of homology. In brief, the CDS of MABC were submitted to CD-HIT-EST [16] with parameters: word length of 5; local sequence identity threshold of 0.8; alignment coverage of 0.8. Only the sequences that passed the set thresholds in two-way comparisons were considered orthologs. Ortholog families containing single representative in all genomes were defined as core families while ortholog families with incomplete representation were defined as accessory families. The clustering output file was analyzed and gene presence/absence binary matrix was constructed. The binary matrix was submitted to PanGP version 1.0.1 [17] for pan-genome profile calculations and visualization.

### Alignment and phylogenetic analysis

To infer the evolutionary relationship among MABC, the single-copy core genes were aligned using MUSCLE version 3.8.31 [18] with default parameters. Orthologous gene families presenting more than one copy from any genome were not included for phylogeny inference to reduce potential phylogenetic noise. The resulting alignments of each individual gene cluster were concatenated and submitted to RaxML version 8.0 [19] for phylogenomic tree inference. ModelTest-NG [20] was used to identify the most appropriate nucleotide substitution model of the alignment.

### Identification of antibiotic resistance proteins

Comprehensive Antibiotic Resistance Database (CARD) version 3.0.7 [21] and Antibiotic Resistance Gene-ANNOTation (ARG-ANNOT) version 4 [22] were used to identify antibiotic resistance protein (ARP) homologs in MABC. All 2,624 and 2,225 ARP sequences were downloaded from CARD and ARG-ANNOT, respectively. Protein sequences in 1,581 strains were subjected to all-against-all BLASTP searches against these two databases with 50% sequence identity and 50% query coverage cut-off. Additionally, the entire putative ARP sequences from MABC were used as TBLASTN queries against the 1,581 MABC genomes (*e*-value threshold of 1 × 10^–5^ with at least 50% query coverage) to identify mis-annotated sequences.

### Tests for selection and recombination

In this study, only ARP ortholog sets that were detected in at least 50 strains per MABC subspecies were analyzed. Codon alignments of orthologous sequences were generated using PAL2NAL software version 14.0 [23], with protein alignments obtained in MUSCLE. To eliminate the impact of recombination on positive selection evaluations, PhiPack version 1.0 [24] was used to infer the presence of recombination in the ARPs. Three recombination-detection statistics: pairwise homoplasy index (PHI), maximum χ^2^ (MaxChi^2^) and neighbor similarity score (NSS) were implemented to test for signatures of recombination. In all three tests, recombination was considered significant if the *p*-value was less than 0.1.

To reveal the selection pressures, Single-Likelihood Ancestor Counting (SLAC) [25], Fixed Effects Likelihood (FEL) [25], Fast Unconstrained Bayesian AppRoximation (FUBAR) [26] and Mixed Effects Model of Evolution (MEME) [27] models which were implemented in Hyphy package [28] were used. SLAC was carried out for the global estimation of the ratios of non-synonymous (*dN*) to synonymous (*dS*) substitution rates (*ω* = *dN/dS*) of the ARPs. The ARPs with *ω* < 1 and *ω* > 1 were inferred to have evolved under purifying selection and diversifying selection, respectively. FEL, FUBAR, MEME and SLAC were also used to detect the sites under diversifying or purifying selection. A significance level of posterior probability (PP) greater than 0.9, *p*-value less than 0.05 and *ω* < 1 or *ω* > 1 were considered as indicating sites under purifying and diversifying selection, respectively. A codon was considered to have undergone purifying or diversifying selection only if it was detected by at least two of the methods implemented.

### Identification of ARP pseudogenes

Pseudogenes were identified by performing TBLASTN searches of all putative MABC ARPs sequences against 1,581 MABC genomes. There is no standard method or criteria to define pseudogene [29]. Most studies [30, 31] focused on sequence identity and e-value as criteria for homology-based pseudogene annotation and there is a hypothesis on the sequence length reduction on pseudogene when compared to its functional gene [32]. Hence in this study, the matching genomic regions with *e*-value below 1 × 10^–5^ and alignment coverages of greater than 15% but less than 50% over the protein sequence queries were considered as pseudogenes.

### Identification of horizontally transferred ARPs

To predict the putative origin of ARPs acquired from horizontal gene transfer events, all MABC ARPs were subjected to similarity search against A CLAssification of Mobile genetic Elements (ACLAME) database version 0.4 [33] with 50% sequence identity and 50% query coverage cut-off.

## Results

### Overview of population

There were 33 complete and 1,548 draft genomes available when this study commenced. The genome sizes ranged from 4.5 M to 6.3 M bps with a mean C + G content of 64.11% (Additional file 1: Table S1). Strain PAP165 (accession number GCF_001214245) was the largest genome. The number of genes per genome ranged from 4,380 to 7,223, with an average of 4,987 CDS. The *hsp65* analysis resulted in 98% to 100% similarities to the *hsp65* in type strain ATCC19977, indicating all strains are the members of MABC. In addition, the pairwise MUM indexes from 0 to 0.36 of all genomes with ATCC19977 further support the species and completeness of the genomes used.

Gene orthology analysis clustered 7,883,753 genes into 61,267 families. The highest number of genes within a single family was 3,915. Paralogs were predicted in 3,213 families but no paralogs were consistently present in all strains. There were 277 core gene families of which, 190 were single-copy. Of the 60,990 accessory gene families, 24,967 were strain-specific. The single-copy genes were used to perform a phylogenetic analysis and the output from gene clustering analyses was utilized to predict the pan-genome in MABC. Although the trend of the pan-genome in MABC has been predicted in previous studies [10, 34], a much larger number of genomes was available in this study to obtain a more accurate prediction. The pan-genome graph showed unbounded expansion with the increase of new genomes to the MABC genome complex (Additional file 2: Fig. S2) with no signs of lowering area under the graph or plateau formation. Hence, the ‘open’ pan-genome of MABC was confirmed in this study.

### Phylogenetic analysis

**Fig. 1:**
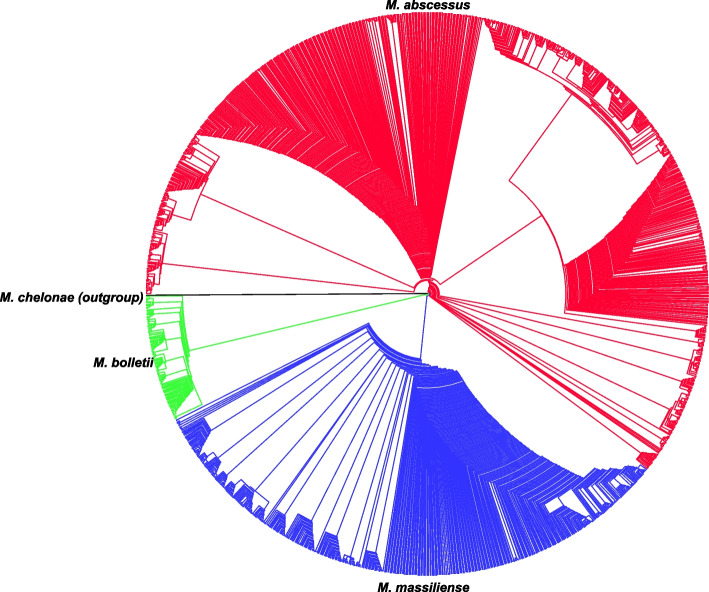
Supermatrix phylogenomic tree. A phylogenomic tree was constructed based on the concatenation of 190 single-copy genes using the maximum likelihood method. The tree classified 1,581 MABC strains into 3 distinct subspecies: *M. abscessus* (red), *M. bolletii* (green) and *M. massiliense* (blue)

The phylogenomic tree of 1,581 MABC strains was constructed using the maximum-likelihood approach implemented in RaxML. A GTR + I + G4 model was selected as a best-fit model for the alignment. Classification based on single-copy core genes using *Mycobacteroides chelonae* as the outgroup showed 961, 115 and 505 strains as *M. abscessus* (red), *M. bolletii* (green) and *M. massiliense* (blue), respectively (Fig. 1).

### Resistance proteins across genomes

**Fig. 2:**
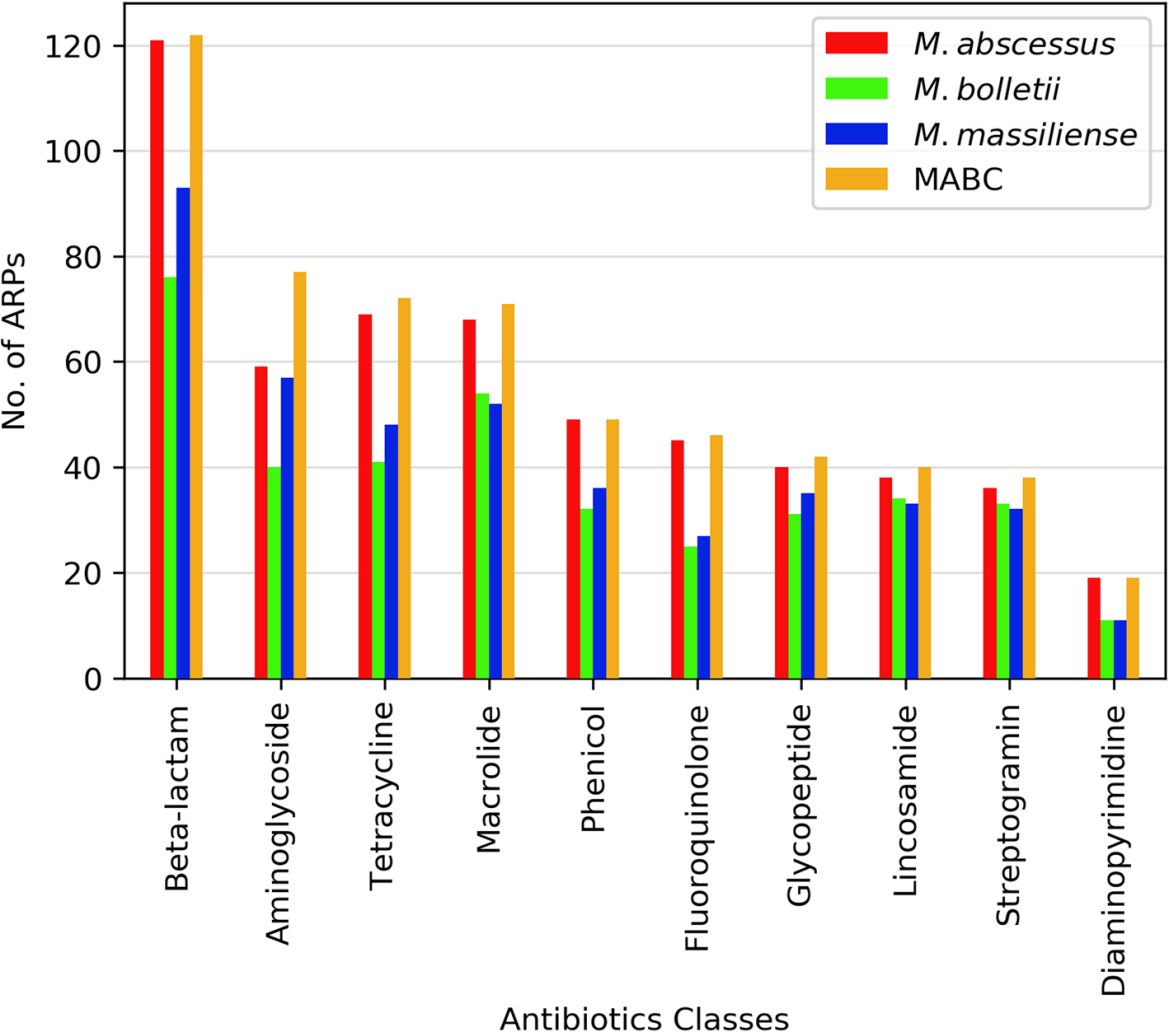
Top 10 antibiotic classes with most abundant ARPs. ARPs were categorized according to the drug classes to which they confer resistance. The top 10 drug classes with most abundant APRs in MABC included beta-lactam, aminoglycoside, tetracycline, macrolide, phenicol, fluoroquinolone, glycopeptide, lincosamide, streptogramin and diaminopyrimidine. For each class, the number of ARPs present in *M. abscessus* (red), *M. bolletii* (green), *M. massiliense* (blue) and MABC (orange) were counted

Antibiotic resistance is a common feature in all three subspecies of MABC. Our search against known ARPs in public databases showed 395 putative MABC ARPs conferring resistance to 28 distinct antibiotic classes, including beta-lactams, aminoglycosides, tetracyclines and others (Fig. 2, Additional file 1: Table S2). Of these 395 MABC ARPs, 303 (77%) ARPs predicted resistance to only one antibiotic class. The number of ARPs in each subspecies varied greatly. The distribution of ARPs is discussed individually, in the following paragraphs.

#### Multidrug resistance

ARPs associated with resistance to more than one antibiotic class were categorized as multiple drug resistant (MDR). These ARPs formed the largest group (92 ARPs, 23.3%) representing resistance to 20 classes of antibacterial agents including antibiotics, acridine dye, benzalkonium chloride, and others (Additional file 1: Table S3). Fifty-four of these ARPs were antibiotic efflux proteins of the ATP-binding cassette (ABC), multidrug and toxic compound extrusion (MATE), major facilitator superfamily (MFS), resistance-nodulation-cell division (RND) and small multidrug resistance (SMR) transporters. Included in this list were the MexAB-OprM and MuxBC-OpmB multiple-membrane component RND antibiotic efflux proteins and the baeSR two-component system regulator of the RND efflux system. Nineteen efflux proteins, adeF, adeJ, arlR, AxyY, baeR, efpA, efrB, KpnH, mdsA, mdsB, MexF, MexW, mtrA, mtrD, MuxB, OpmD, smeD, smeE and smeR were detected in more than 1,500 MABC strains but others differed in their distribution among the three MABC subspecies. For instance, mdtF was frequently observed in *M. abscessus* (927 strains) but was only observed in 8 *M. bolletii* and 89 *M. massiliense* strains, while golS and mdsC were more prevalent in *M. massiliense* (< 170 strains) compared to *M. abscessus* (< 110 strains), and *M. bolletii* (< 15 strains).

Besides efflux proteins, the ABC-F ATP-binding cassette ribosomal protection proteins (RPPs), excluding lsaB and poxtA, were also prevalently detected in MABC (> 1,575 strains). In addition, fourteen 23S rRNA methyltransferases (Erm(33), Erm(43), Erm(44), Erm(45), Erm(46), Erm(48), ErmA, ErmB, ErmC, ErmG, ErmH, ErmO, ErmT and ErmY) were also abundant. A beta-lactamase, blaLAP-1 which is also responsible for fluoroquinolone, aminoglycoside, tetracycline and rifamycin resistance [35], was detected in all but one (1,580) strain. In contrast, another beta-lactamase blaTLA-2 which is associated with beta-lactam and fluoroquinolone resistance [36] was only detected in 1 *M. abscessus* strain. Altogether, *M. abscessus* was the subspecies with the most (89) ARPs associated with MDR and *M. massiliense* was found to have the least (61) ARPs.

#### Beta-lactam antibiotics

The antibiotic class with the second highest number of ARPs comprised the beta-lactam antibiotics, with 90 (22.7%) putative ARPs involved in resistance to carbapenems, cephalosporins, cephamycins, monobactams, and other beta-lactam antibiotics (Additional file 1: Table S4). Of these 90 ARPs, 83 were grouped into 61 beta-lactamase families. The remaining 7 ARPs were families of general bacterial porins with reduced permeability to beta-lactams, penicillin-binding proteins (PBP1 and PBP2), regulatory proteins and repressor proteins.

Among the proteins responsible for resistance to carbapenems and cephamycins were 19 beta-lactamase families (ADC, AIM, BKC, Bla, BlaB, CAU, CMY, CphA, DHA, GES, IND, KPC, MOX, OXA, PEDO, PNGM, SHV, THIN-B and VIM) and a porin protein (Omp38). Of these, blaAIM-1, blaB-4, blaCAU-1, blaIND-6, blaKPC-11, blaOXA-117, blaOXA-455, blaOXA-60, blaOXA-666, blaSHV-1 and blaVIM-7 were frequently detected in all subspecies (> 1,575 MABC strains). However, the blaADC-16 protein was more frequently observed in *M. abscessus* (633 *M. abscessus*, 38 *M. bolletii* and 7 *M. massiliense* strains) while blaCMY-1 and cepH were more prevalently detected in *M. massiliense* (166 *M. massiliense*, 91 *M. abscessus* and 9 *M. bolletii* strains).

Among the three subspecies, while *M. abscessus* had all 90 ARPs, there were only 77 and 60 in *M. massiliense* and *M. bolletii* respectively. On the other hand, *M. bolletii* had the most shared ARPs (47 ARPs) that were present in all members of this subspecies, followed by *M. massiliense* (45 shared ARPs) and *M. abscessus* (8 shared ARPs).

#### Aminoglycosides

For aminoglycosides, 63 (15.9%) putative ARPs associated with resistance were detected. They were various types of acetyltransferases (24), phosphotransferases (16), 16S rRNA methyltransferases (4), nucleotidyltransferases (16), one bifunctional aminoglycoside-modifying enzyme (ANT(3″)-Li-AAC(6’)-IId) and two RND efflux proteins (acrD, amrB) (Additional file 1: Table S5). Eight acetyltransferases (AAC(2')-Ib, AAC(2')-Ic, AAC(2')-Id, AAC(2')-Ie, AAC(3)-IIIa, AAC(3)-VIa, AAC(6')-IIa and aacA43), three 16S rRNA methyltransferases (kamB, kdpE and rmtH), two nucleotidyltransferases (ANT(2'')-Ia and ANT(4')-Ia) and most of the phosphotransferases (excluding APH(6)-Ia, APH(6)-Id, APH(7'')-Ia and aph4-Ib) were frequently distributed among the strains (> 1,500 strains). The bifunctional enzyme (ANT(3'')-Ii-AAC(6')-IId) was only detected in 4 *M. massiliense* strains. Overall, *M. massiliense* (51 ARPs) appeared to have the most aminoglycoside-inactivating enzymes, followed by *M. abscessus* (43 ARPs) and *M. bolletii* (33 ARPs).

Amikacin, the aminoglycoside that is resistant to most aminoglycoside-modifying enzymes, is frequently used in the treatment of MABC infections. The transferases associated with amikacin resistance were methyltransferases (rmtD, and rmtH), acetyltransferases (AAC(3)-IIa and AAC(6')-Ib), nucleotidyltransferases (ANT(2'')-Ia and ANT(4')-Ia), and phosphotransferases (APH(3')-IIa and aphA2). The nucleotidyltransferases and phosphotransferases were found in almost all (1,575) MABC strains, indicating a genetic predisposition to phenotypic amikacin resistance in all MABC. In contrast, AAC(3)-IIa was observed in less than half of the MABC strains (336 M*. abscessus*, 297 *M. massiliense* and 3 *M. bolletii* strains) while AAC(6')-Ib was only identified in 4 *M. massiliense* strains.

#### Glycopeptides

ARPs associated with glycopeptide resistance formed 10.6% of total ARPs (Additional file 1: Table S6). They consisted of 14 van ligases associated with 11 vancomycin resistance phenotypes, as well as 28 van proteins assigned to 9 classes of glycopeptide resistance gene clusters. Twelve van ligases (vanA, vanA-Ao2, vanB, vanC, vanC2/3, vanD, vanE, vanG, vanI, vanL, vanM and vanN) and 7 van proteins (vanH-Ac2, vanO, vanRF, vanRI, vanRM, vanSA and vanS-Pt) were observed in all MABC strains and vanHO, vanKI, vanRB, vanRL, vanTG, vanTN, vanTrL, vanX-Pt2, vanX-Sc, vanAE-Pp and vanF were frequently observed in > 1,500 strains. Overall, *M. abscessus* appeared to have the highest number of ARPs, with vanSG and vanSL being present exclusively in this subspecies (Additional file 1: Table S6).

#### Tetracyclines

The distribution of 28 (7.1%) ARPs associated with tetracycline resistance is shown in Additional file 1: Table S7. They comprised efflux proteins (MFS, ABC and RND), ribosomal protection protein (RPP) and tetracycline-inactivating enzymes. Those frequently observed (in over 1,570 strains) and present in all three MABC subspecies were the efflux proteins otrC, tetB(60), tap, tet(41), tetA(48), tetE, tetJ, tetR(G), tetV, tetZ; the RPP otr; and inactivating enzymes tet(49) and tet(55) [37]. Less frequently seen were inactivating enzymes tet(50), tet(52) and tet(56) which were only detected in 407 *M. massiliense* and 86 *M. abscessus* strains. The rarely observed ARPs were the efflux genes tet(45), tetB, tetH, tetK, tetL, adeB and the tetracycline monooxygenase tetX which were observed in less than 5 *M. abscessus* strains, as well as the adeC, tet(33) and tet(40) efflux pumps which were predicted in only two *M. massiliense* strains. Overall, *M. massiliense* had the most (12) shared ARPs that were present in all members of this subspecies, followed by *M. bolletii* (11 shared ARPs) and *M. abscessus* (6 shared ARPs).

#### Phenicols

There were 13 (3.3%) ARPs related to phenicols in *M. abscessus*, 12 in *M. massiliense* and 9 in *M. bolletii* (Additional file 1: Table S8). The acetyltransferase, catA5, was in all strains while four other acetyltransferases (cat86, catA4, catI and catP) were frequently observed in all three subspecies. Similarly, three MFS efflux proteins (cml, cmr and fexA) were prevalently detected in MABC (> 1,575 strains) but dha1 was observed in less than 10 MABC strains. Three other efflux proteins (floR, pp-flo and mexN) were each detected in less than 10 *M. abscessus* and *M. massiliense* strains. The cmx ARP was only observed in one *M. abscessus* strain.

#### Fluoroquinolones

There were 11 putative ARPs (2.8%) involved in fluoroquinolone resistance (Additional file 1: Table S9), 10 of which were efflux proteins (patB, AbaQ, emrA, emrB, lrfA, norB, qacA, qacB, qepA and qacH). With the exception of AbaQ, emrA, norB and qacH, the efflux proteins were detected in all subspecies (≥ 1,579 MABC strains). AbaQ and norB were the two least frequent ARPs, being detected in only 1 *M. abscessus* and 5 *M. massiliense* strains respectively. The emrA proteins were observed in 239 *M. abscessus* and 25* M. massiliense* strains but not in *M. bolletii*. The one remaining ARP, protein mfpA which binds to and protects DNA gyrase from quinolones [38] was observed in all strains.

#### Other antibiotic classes

There were 19 classes of antibacterial agents with less than 10 ARPs each. These were the acridine dyes, aminocoumarin, bicyclomycin, diaminopyrimidines, elfamycin, fosfomycin, fusidic acid, lincosamides, macrolides, mupirocin, nitroimidazole, nucleoside, peptides, pleuromutilin, rifamycin, streptogramin, sulfonamide, tetracenomycin and triclosan. The distribution of these ARPs in MABC subspecies is listed in Additional file 1: Table S10. Thirteen of these ARPs were widely found in MABC (> 1,500 strains) but three phosphoethanolamine transferases responsible for colistin resistance were found in < 500 strains, four thiol transferases and a phosphotransferase associated with fosfomycin and peptide resistance, respectively, were predicted only in *M. massiliense*, and two ARPs associated with aminocoumarin resistance were detected only in *M. abscessus*.

### Recombination and selection analyses

Before studying phylogenetic relatedness and evolutionary pressure on MABC resistomes, recombination in ARPs was first excluded as recombination can confound a selection analysis by causing mutation rate heterogeneity among sites [39]. After recombination analysis, 14 ARPs (arr-8, Erm(33), Erm(43), Erm(44), Erm(45), Erm(48), ErmA, ErmB, ErmC, ErmG, ErmT, ErmY, sul1 and sul2) were found to show recombination signals (homoplasies, recombination breakpoints and non-uniform distribution of sequence variations in alignments) in all three recombination detection methods (Additional file 1: Table S11) and were excluded from selection analysis.

In evolutionary biology, diversifying selection favors amino acid changes (nonsynonymous mutations) that alter protein structures, whereas purifying selection removes amino acid changes to stabilize proteins [40]. In this study, an excess of synonymous mutations over nonsynonymous mutations (*ω* < 1) was detected in 202 ARPs by SLAC. This suggested that purifying selection is the principal evolutionary force shaping diversity at these proteins (Additional file 1: Table S12). Of the 202 putative ARPs under purifying selection, 49 were beta-lactamases, 47 efflux proteins (7 ABC, 1 MATE, 22 MFS and 17 RND efflux proteins), 39 transferases (14 acetyl-, 3 ADP-ribosyl-, 1 glycosyl-, 5 methyl-, 2 nucleotidyl- and 14 phospho- transferases), 16 van proteins of different clusters, 14 van ligases, 13 ABC-F ATP-binding Cassette RPPs, 8 dihydrofolate reductases, 3 isoleucyl-tRNA synthetases, 2 beta-subunits of RNA polymerase, 2 tetracycline-inactivating enzymes, an aminocoumarin resistant protein, a fusidic acid-inactivating enzyme, a PBP2, a quinolone resistance protein, an RNA polymerase-binding protein, a RPP, a monooxygenase, a dihydropteroate synthase and lastly a tetracenomycin C resistance and export protein. In contrast, a macrolide esterase (EreA) and a methyltransferase (ErmO) were predicted to have undergone diversifying selection by a higher number of nonsynonymous mutations than synonymous mutations (*ω* > 1) (Additional file 1: Table S12).

In addition to protein-wide selection, all proteins were further assessed for site-wise selection with FEL, FUBAR, MEME and SLAC models implemented in Hyphy (Additional file 1: Table S12). Different than EreA and ErmO which showed higher protein-wide nonsynonymous mutations than synonymous mutations, APH(3'')-Ic, efrB, lrfA and tap were identified with diversifying selection at selective sites (Additional file 1: Table S12). Two sites in APH(3″)-Ic and 1 site each in erfB, lrfA and tap were predicted to experience diversifying selection. Of the remaining ARPs, parY was the protein with the highest number of sites under purifying selection (260 sites detected by more than two methods).

### ARPs Acquisition in MABC

**Fig. 3:**
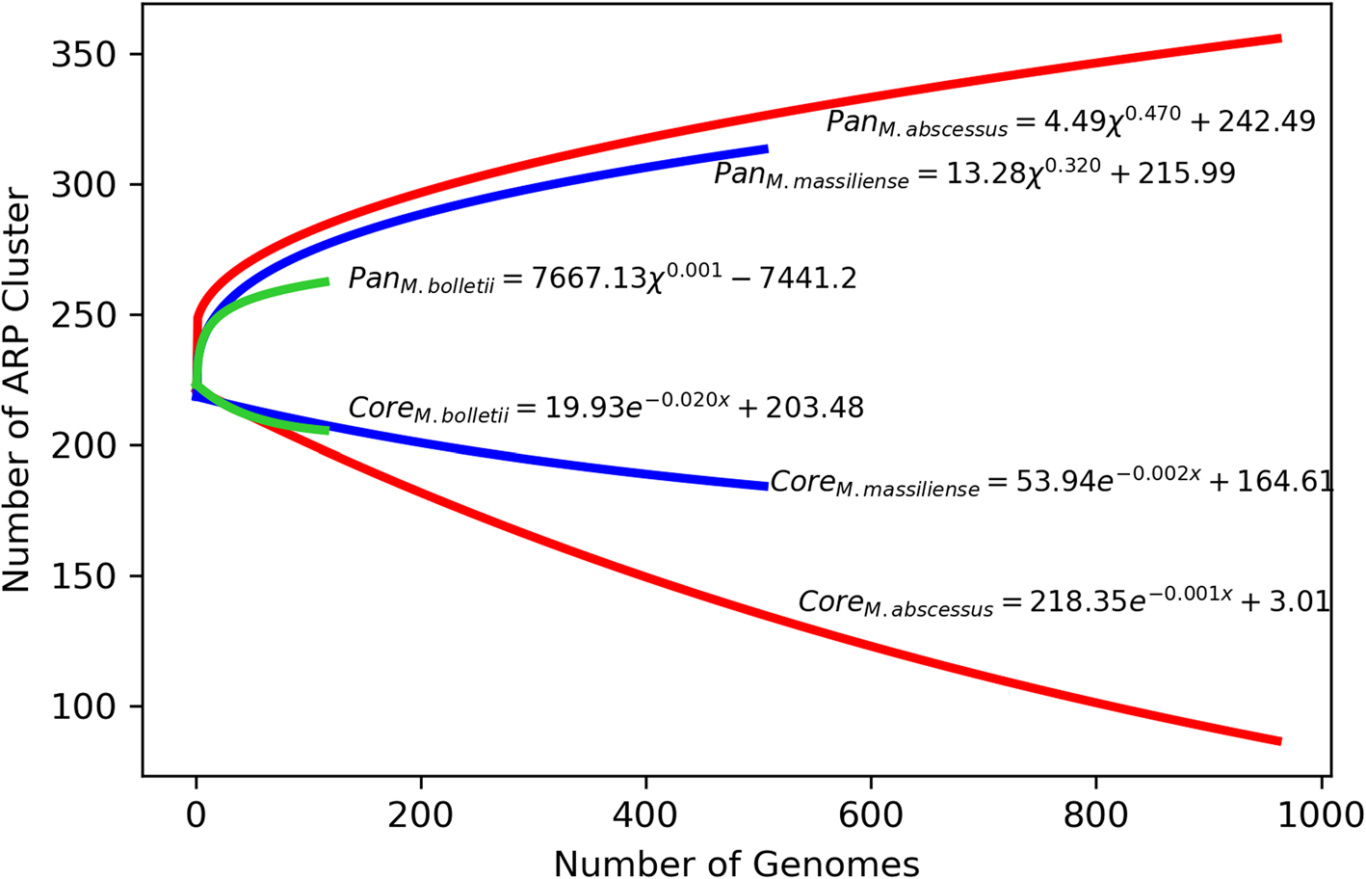
The core- and pan-resistome plot of MABC subspecies. The core- and pan-resistome curves of the three subspecies are indicated using red (*M. abscessus*), green (*M. bolletii*) and blue (*M. massiliense*). The core-resistome curves are the least-squares fit of exponential decay function to average values of data, whereas the pan-resistome curves are a least-squares fit of the power-law to average values. The pan-resistome curve’s exponent > 0 indicates an open pan-resistome in the subspecies

We analyzed trends of ARPs in the MABC population with the pan-resistome approach. If resistomes in the MABC complex were the result of vertical evolution, the distribution of ARPs would reflect minimal or no changes in the core genome plots. However, we observed that, with the introduction of each new genome, the core-resistome decreased while the accessory resistome increased (Fig. 3). Hence, the gain of new genetic material appeared to cause a restructuring of the core and accessory resistomes. We hypothesized that the uneven distribution of the ARPs in MABC could be related to gene gain via horizontal gene transfer (HGT) or gene loss by pseudogenization processes. To check for pseudogenes, all MABC ARP sequences were served as input queries to TBLASTN against MABC genomes. The results showed only 18 ARPs to have undergone a pseudogenization event (Additional file 1: Table S13) of which, 12 were observed in less than 5 MABC strains. Two pseudogenized methyltransferases, Erm(37) and Erm(41), were identified in 496 out of 505 *M. massiliense* strains. Among *M. bolletii*, 13 strains were predicted to have pseudogenized kamB, 8 strains with ANT(4’)-Ia and 8 strains with tet(49). Pseudogenized ARPs (AAC(6'), AAC(6')-Isa, blapenA, mecI, blaI, acrA and tet(45)) were detected in *M. abscessus* strains. From these figures, pseudogenization appeared to be an insignificant cause of the uneven distribution of MABC ARPs.

HGT facilitates the dissemination of genetic material between bacteria via mobile genetic elements (MGEs) such as plasmids, transposons, viruses and prophages [41]. In this study, 285 out of 395 (72.2%) MABC ARPs were found in the ACLAME database of MGEs (Additional file 1: Table S14). These MGEs were mainly plasmids (229), prophages (26) and viruses (14). The ARPs involved included 16 that were homologous to two different MGEs, 82 efflux proteins (44 RND, 25 MFS, 10 ABC, 2 SMR, and 1 MFS and RND), 64 transferases (17 acetyl-, 15 methyl-, 15 nucleotidyl-, 14 phospho-, 2 phosphoethanolamine- and 1 nucleotidyl- and acetyl- transferases), 64 beta-lactamases, 16 van proteins of various clusters, 16 ABC-F ATP-binding cassette RPPs, 14 van ligases, 8 dihydrofolate reductases, 6 tetracycline-inactivating enzymes, 3 isoleucyl-tRNA synthetases, 2 PBP2, 2 beta-subunits of RNA polymerase, 2 dihydropteroate synthases and others (one each of regulatory protein, repressor protein, fusidic acid-inactivating enzyme, monooxygenase, RPP and tetracenomycin C resistance and export protein). The abundance of MGEs, particularly in the ARPs of the accessories genomes (222 out of 285) likely played an important role in accelerating HGT events in MABC.

## Discussion

MABC is an opportunistic pathogen of increasing medical concern. The treatment of MABC infections is difficult due to its intrinsic resistance to a wide range of antimicrobial drugs. Moreover, resistance can be acquired via horizontal gene transfer (HGT). In this study, the core- and pan-genome plot (Additional file 2: Fig. S2) suggested that MABC has an ‘open’ pan-genome which, aided by HGT, will continue to acquire new genes into its gene pool. This is consistent with earlier findings of pan-genome studies performed on mycobacterial species [42–44].

It is not clear how the antibiotic resistances of MABC are related to the presence of ARPs, especially those in the accessory genomes. The ARPs identified in this study were predicted to be highly associated with multi-drug resistance (MDR). The dominance of efflux proteins as a cause of MDR is evidenced by the large number of ARPs linked to the efflux mechanism, such as the MexAB-OprM with its regulator CpxR that is known to be involved in resistance to multiple antibiotics [45]. The high number of ARPs associated with resistance to beta-lactam antibiotics is expected as MABC has been reported to be intrinsically resistant to all beta-lactams except carbapenems and cephamycins and hydrolysing beta-lactamases have been considered the major mechanism causing resistance to a broad spectrum of beta-lactams in MABC. The results from this study corroborated the more important role of hydrolysing beta-lactamases over altered penicillin-binding proteins in the resistance of MABC to beta-lactams, including carbapenems and carbamycins. Similarly, the results supported previous reports that the aminoglycoside-inactivating enzymes are the major mechanism of aminoglycoside resistance in MABC [46, 47] and that broad-spectrum aminoglycoside resistance is likely due to bifunctional aminoglycoside-modifying enzymes. As expected, resistance to fluoroquinolones is most likely due to the efflux proteins and quinolone resistance protein found widely prevalent among the MABC in this study. Consistent with previous findings [48, 49], trimethoprim resistance in MABC is probably caused by dihydrofolate reductases (DHFR). Some interesting features noted among the less prevalent ARPs predicted are: 1) the occurrence of tetX reported by Luthra et al. [46] to be responsible for high level tetracycline resistance in MABC, 2) the plasmid-borne resistance factors in colistin, the last-resort antibiotic for the treatment of MDR infections and 3) the RND efflux pump (LpeAB) which is a homolog of the efflux protein in *Legionella pneumophila* reported by Massip et al. [50] to be associated with high macrolide resistance in *L. pneumophila*. Overall, *M. abscessus* was found to be the subspecies with the most resistance-associated proteins, followed by *M. massiliense* and *M. bolletii.*

In this study, one Arr, eleven Erm and two Sul proteins were predicted to have undergone recombination. The *arr*, *erm* and *sul* genes are usually associated with mobile genetic elements such as plasmids, integrons and transposons, which facilitate the dissemination of antibiotic resistance through HGT [51–53]. Our results are consistent with those in earlier studies [54, 55] that reported Erm proteins to be frequently involved in HGT in gram-positive bacteria. This observation was also reported in the phylogenetic analysis of methyltransferases conducted by Park et al. [56], which showed the phylogenetic incongruence in the Erm clade to be caused by HGT and gene duplication events.

In the search for evolutionary pressures based on the number of synonymous mutations and non-synonymous mutations, the ARPs in MABC were observed to be exerting purifying rather diversifying selection. This suggests the MABC is achieving stability by reducing the diversity of ARPs. On the other hand, most of the ARPs (81.5%) were observed in the accessory genomes, and 72.2% of ARPs were associated with mobile genetic elements involved in HGT, a process that increases foreign genetic material in microbial cells. Pseudogenization of the ARPs could also increase ARP diversity, but we predicted these events to be infrequent in MABC, except for the truncation in Erm(37) and Erm(41) that is known to be a regular feature in *M massiliense* [57–59]. These observations suggest HGT to be the main source of ARPs in MABC, particularly in the MABC accessory genome.

## Conclusion

This in silico prediction of resistomes in MABC gives a bird’s eye view of possible resistance mechanisms that could affect the choice of antibiotic therapies for MABC infections. Uneven resistome profiles were predicted across MABC strains and subspecies. The shared ARPs in MABC could be in stable condition because of the purifying selection in MABC that does not allow diversification through significant amino acid changes. Coupled with mobile genetic element and pseudogene analyses, the examination of more than 1,500 genome sequences yielded data suggesting horizontal gene transfer as the main contributor to the structure of the pan-genome, particularly the resistome, of MABC. Hence, the multidrug resistance problem with this bacterium is expected to become more serious with time.

## Supplementary Information

**Additional file 1:** **Table S1.**General Information on MABC Genomes. **Table S2.** Amount of Antibiotic Resistance Proteins (ARPs) Detected in MABCper Antibiotic Classes. **Table S3.**Distribution of Antibiotic Resistance Proteins (ARPs) Associated withResistance to Multiple Antibiotic Classes. **Table S4.** Distribution ofAntibiotic Resistance Proteins (ARPs) Associated with Beta-lactam Resistance. **TableS5.** Distribution ofAntibiotic Resistance Proteins (ARPs) Associated with AminoglycosideResistance. **Table S6.** Distribution of Antibiotic Resistance Proteins(ARPs) Associated with Glycopeptide Resistance. **Table S7.** Distributionof Antibiotic Resistance Proteins (ARPs) Associated with TetracyclineResistance. **Table S8.** Distribution of Antibiotic Resistance Proteins(ARPs) Associated with Phenicol Resistance. **Table S9.** Distribution ofAntibiotic Resistance Proteins (ARPs) Associated with FluoroquinoloneResistance. **Table S10.** Distributionof Antibiotic Resistance Proteins (ARPs) Associated with Resistance to OtherAntibiotic Classes. **Table S11.** Detailed Information on Test ofRecombination. **Table S12.** List of Antibiotic Resistance Proteins (ARPs)that Underwent Selection. **Table S13.** Distribution of AntibioticResistance Proteins (ARPs) Pseudogene in MABC. **Table S14.** List ofAntibiotic Resistance Proteins (ARPs) with Homologs in ACLAME.

**Additional file 2:** **Fig.S1** Flowchart describing the entire workflow. Anoverview of the main methods and results in the current study. **Fig. S2** The core- and pan-genome plotof studied MABC subspecies. The core- and pan-genome curves of the three MABCsubspecies were illustrated using red (*M.abscessus*), green (*M. bolletii*)and blue (*M. massiliense*). Thecore-genome curve represents the least-squares fit of exponential decayfunction to average number of gene families that consistently observed for eachaddition of new genomes, whereas the pan-genome curve indicate the power lawfitting of the average number of novel gene family added per additional genomesequences. The exponents (0.44 in *M.abscessus*, 0.30 in *M. bolletii*and 0.42 in *M. massiliense*) ofpan-genome curves are greater than zero, indicating each MABC subspecies has anopen pan-genome.

## Data Availability

The genome sequences of 1,581 Mycobacteroides abscessus complex used in this study are available in the NCBI RefSeq database (https://www.ncbi.nlm.nih.gov/refseq/). The accession numbers of all sequences are shown in Additional file 1: Table S1.

## Abbreviations

ABC: ATP-binding cassette
ACLAME: A CLAssification of Mobile genetic Elements
ARG-ANNOT: Antibiotic Resistance Gene-ANNOTation
ARP: Antibiotic resistance protein
CARD: Comprehensive Antibiotic Resistance Database
CDS: Coding sequences
DHFR: Dihydrofolate reductases
dN/dS: Non-synonymous to synonymous substitution rates
FEL: Fixed Effects Likelihood
FUBAR: Fast Unconstrained Bayesian AppRoximation
HGT: Horizontal gene transfer
MABC: *Mycobacteroides abscessus* complex
MATE: Multidrug and toxic compound extrusion
MaxChi^2^: Maximum χ^2^
MDR: Multi-drug resistance
MEME: Mixed Effects Model of Evolution
MFS: Major facilitator superfamily
MGE: Mobile genetic elements
MTBC: *Mycobacterium tuberculosis* complex
NCBI: National Center for Biotechnology Information
NSS: Neighbor similarity score
NTM: Nontuberculous mycobacteria
PHI: Pairwise homoplasy index
RND: Resistance-nodulation-cell division
SLAC: Single-Likelihood Ancestor Counting
SMR: Small multidrug resistance
WGS: Whole genome sequencing
