# Supplementary material for: The resistomes of Mycobacteroides abscessus complex and their possible acquisition from horizontal gene transfer
Source: BMC Genomics. 2022 Oct 20;23:715. doi: 10.1186/s12864-022-08941-7 (PMC9583574; doi:10.1186/s12864-022-08941-7)
Supplement: Supplementary file 2 — Additional file 2: Fig.S1 Flowchart describing the entire workflow. Anoverview of the main methods and results in the current study. Fig. S2 The core- and pan-genome plotof studied MABC subspecies. The core- and pan-genome curves of the three MABCsubspecies were illustrated using red (M.abscessus), green (M. bolletii)and blue (M. massiliense). Thecore-genome curve represents the least-squares fit of exponential decayfunction to average number of gene families that consistently observed for eachaddition of new genomes, whereas the pan-genome curve indicate the power lawfitting of the average number of novel gene family added per additional genomesequences. The exponents (0.44 in M.abscessus, 0.30 in M. bolletiiand 0.42 in M. massiliense) ofpan-genome curves are greater than zero, indicating each MABC subspecies has anopen pan-genome. [file 12864_2022_8941_MOESM2_ESM.docx]

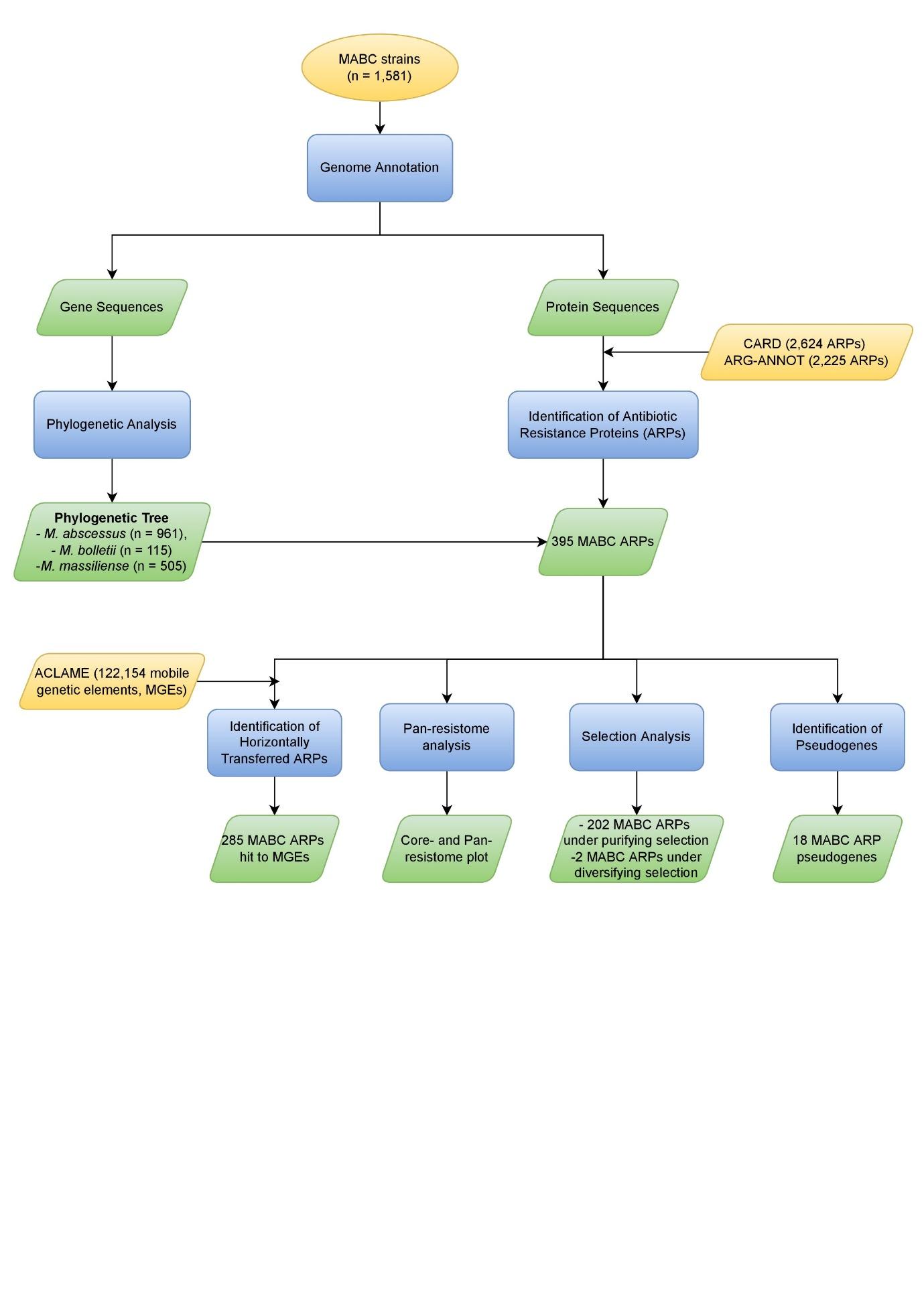


**Fig. S1. Flowchart describing the entire workflow.**  An overview of the main methods and results in the current study.


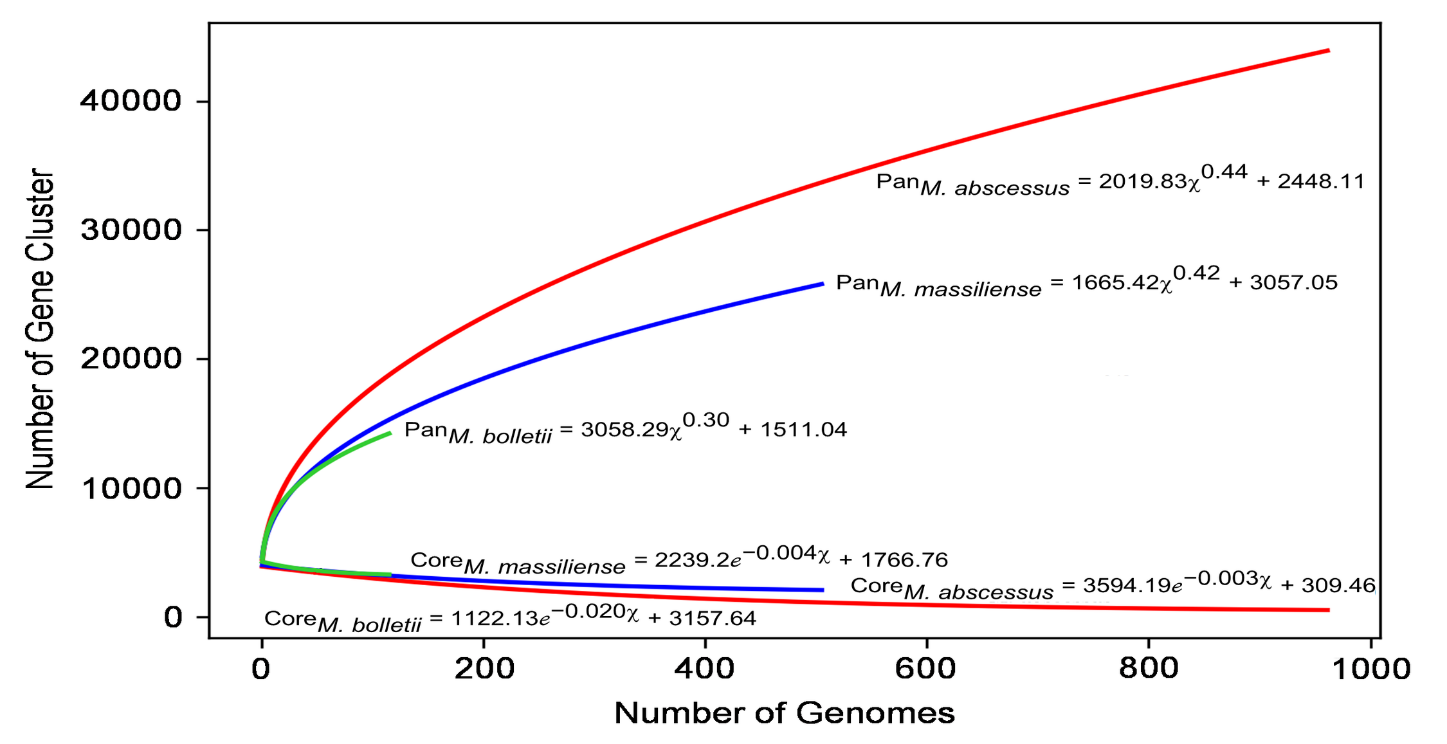


# Fig. S2. The core- and pan-genome plot of studied MABC subspecies. The core- and pan-genome curves of the three MABC subspecies were illustrated using red (*M. abscessus*), green (*M. bolletii*) and blue (*M. massiliense*). The core-genome curve represents the least-squares fit of exponential decay function to average number of gene families that consistently observed for each addition of new genomes, whereas the pan-genome curve indicate the power law fitting of the average number of novel gene family added per additional genome sequences. The exponents (0.44 in *M. abscessus*, 0.30 in *M. bolletii* and 0.42 in *M. massiliense*) of pan-genome curves are greater than zero, indicating each MABC subspecies has an open pan-genome.
